# Exploring the serotonin‐probiotics‐gut health axis: A review of current evidence and potential mechanisms

**DOI:** 10.1002/fsn3.3826

**Published:** 2023-12-07

**Authors:** Noor Akram, Zargham Faisal, Rushba Irfan, Yasir Abbas Shah, Syeda Ayesha Batool, Toobaa Zahid, Aqsa Zulfiqar, Areeja Fatima, Qudsia Jahan, Hira Tariq, Farhan Saeed, Aftab Ahmed, Aasma Asghar, Huda Ateeq, Muhammad Afzaal, Mahbubur Rahman Khan

**Affiliations:** ^1^ Department of Food and Nutrition Government College University Faisalabad Faisalabad Pakistan; ^2^ Department of Human Nutrition Bahauddin Zakariya University Multan Multan Pakistan; ^3^ Faculty of Food Nutrition & Home Sciences University of Agriculture Faisalabad Pakistan; ^4^ Natural & Medical Science Research Center University of Nizwa Nizwa Oman; ^5^ Department of Food Science Government College University Faisalabad Faisalabad Pakistan; ^6^ National Institute of Food Science & Technology University of Agriculture Faisalabad Pakistan; ^7^ Department of Nutritional Sciences Government College University Faisalabad Faisalabad Pakistan; ^8^ Department of Food Processing and Preservation Hajee Mohammad Danesh Science & Technology University Dinajpur Bangladesh

**Keywords:** GI disorders, gut microbiota, neurotransmitter, probiotic, serotonin

## Abstract

Modulatory effects of serotonin (5‐Hydroxytryptamine [5‐HT]) have been seen in hepatic, neurological/psychiatric, and gastrointestinal (GI) disorders. Probiotics are live microorganisms that confer health benefits to their host. Recent research has suggested that probiotics can promote serotonin signaling, a crucial pathway in the regulation of mood, cognition, and other physiological processes. Reviewing the literature, we find that peripheral serotonin increases nutrient uptake and storage, regulates the composition of the gut microbiota, and is involved in mediating neuronal disorders. This review explores the mechanisms underlying the probiotic‐mediated increase in serotonin signaling, highlighting the role of gut microbiota in the regulation of serotonin production and the modulation of neurotransmitter receptors. Additionally, this review discusses the potential clinical implications of probiotics as a therapeutic strategy for disorders associated with altered serotonin signaling, such as GI and neurological disorders. Overall, this review demonstrates the potential of probiotics as a promising avenue for the treatment of serotonin‐related disorders and signaling of serotonin.

## Graphical abstract

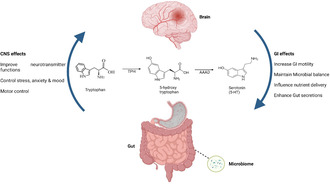

## 1 INTRODUCTION

Serotonin, also known as 5‐Hydroxytryptamine (5‐HT), serves as a crucial neurotransmitter and hormone present in platelets, playing vital roles in both the peripheral nervous system (PNS) and the central nervous system (CNS). Although approximately 90% of the body's serotonin is located in the gastrointestinal tract (GIT), while 10% can be found in the CNS, the neurotransmitter is widely recognized for its significant involvement in the development and progression of various neurological disorders (Guzel & Mirowska‐Guzel, 2022). Serotonin exerts a profound influence on both brain and GI functions; moreover, within the GI tract, a specialized type of cells known as enterochromaffin cells (ECs) serve as endocrine cells responsible for storing approximately 90% of the body's serotonin. These ECs release serotonin in response to various mechanical or chemical stimuli, making them pivotal players in the intricate processes of neurotransmission and serotonin release. Consequently, the activity of ECs contributes significantly to the regulation of serotonin levels in the gut, which in turn plays a crucial role in modulating gut function and its connection to the central nervous system (Stasi et al., 2014).

Specialized serotonin transporter (SERT) absorbs serotonin delivered into the bloodstream and stores it in the thick granules of the platelets (Lesurtel et al., 2012). The SERT is a protein that plays a crucial role in metabolism and is responsible for the reuptake of serotonin from the extracellular space back into nerve cells, plays a crucial role in maintaining serotonin levels in the extracellular space at relatively constant levels (Inoue et al., 2002). However, serotonin is unable to cross the blood–brain barrier; therefore, it must be synthesized de novo inside the serotonergic neurons of the central nervous system; although, tryptophan (TRP) is an essential amino acid and a substrate to several physiologically vital chemicals, including 5‐HT/Serotonin. Since serotonin cannot cross the blood–brain barrier, it is a substrate for several chemicals that are physiologically vital for host metabolism (Liu, Sun, et al., 2021; Papadimas et al., 2012).

Despite the fact that serotonin (5‐HT) is necessary for the regulation of visceral pain and secretion as well as the onset of the peristaltic reflex, abnormal serotonin levels have also been found to cause a variety of psychiatric illnesses. Serotonin interacts with a variety of receptor subtypes to carry out its biological activity. Numerous 5‐HT receptor subtypes are expressed in the gut and affect digestive functions (Bellini et al., 2016; Stasi et al., 2014). Serotonin has the potential to stimulate neurons that are responsible for nerve damage, strengthen the motility of the muscles in the GIT, cause the muscles of the lungs and uterus to compress, affect the vascular muscles in both directions (constriction and relaxation), take part in platelet aggregation, and affect neurons in the central nervous system. Serotonin can function in either a pro‐ or anti‐inflammatory manner, depending on the pathway it takes, and it contributes to the symptoms of GI inflammation (Guzel & Mirowska‐Guzel, 2022).

It is imperative that probiotics are genetically unaltered and robust enough to endure travel through the GIT. Therefore, it might be possible for probiotics to alter the composition and equilibrium of the microbiota that live on the mucosal surfaces of the intestines, particularly the lumen, as probiotics have the ability to suppress the inflammation caused by gut microbes, it has already been demonstrated that probiotics can have a role in lowering inflammatory reactions in GIT, such as those caused by autoimmune illnesses (Mahesh et al., 2021).

Although, gut bacteria play a role in the prevention of mental disorders such as major depressive disorder, anxiety disorders, and trauma‐related diseases. Alterations in gut microbiota may regulate brain‐gut signaling, changing how the cortex responds to neuroendocrine and immunological stimuli, and comorbidity between GI and mental health disorders corroborated these findings (Settanni et al., 2021). Considering this, the human GIT is home to a diverse bacterial community that controls the generation of multiple signaling molecules in the host. These signaling molecules include serotonin or 5‐HT, as well as hormones and neurotransmitters (Noble et al., 2017; Sanders et al., 2019).

However, depression and anxiety disorders are significant contributors to the global nonfatal health burden and are typically managed with pharmaceutical treatments that come with various unwanted side effects. Over the last 10 years, mounting evidence has indicated that the consumption of specific probiotic strains can offer health advantages that extend beyond the digestive system. These probiotics, which can enhance mental well‐being when ingested in the right quantities, have been coined “psychobiotics”, hence, presenting a hopeful complementary method for enhancing the quality of life for individuals experiencing mental distress (Hernandez‐Barrueta, 2022).

Serotonin is a phylogenetically old biogenic amine that maintains energy balance. New research has revealed that peripheral serotonin boosts nutrition absorption and storage. We reviewed current knowledge of peripheral serotonin neurotransmission, focusing on critical effects on GIT function on serotonin metabolism and probiotics that increase serotonin neurotransmission.

## 2 SYNTHESIS AND METABOLISM OF SEROTONIN

Serotonin has contributed to the control of metabolism across various biological groups. Outside the central and enteric nervous systems (ENS), the production of serotonin relies on the enzyme called tryptophan hydroxylase 1 in peripheral tissues (Chen et al., 2021; Stasi et al., 2021). In addition to the neuronal exchanges that occur in the CNS and the ENS, serotonin also affects the tissues of the body's periphery. Moreover, serotonin is responsible for mediating a wide variety of non‐neuronal processes and functions, such as the regulation of the bladder, hemostasis, the respiratory drive, immunological response, vascular tone, and intestinal inflammation (Li et al., 2011).

Central serotonin plays a crucial role in regulating both mood and behavior, as well as influencing the overall energy balance by reducing appetite. Several medications that modulate central serotonin function (such as fenfluramine, sibutramine, and lorcaserin) were initially approved and utilized as anti‐obesity treatments but were subsequently withdrawn due to their adverse cardiovascular and carcinogenic effects. In the past decade, there has been extensive research into the role of peripheral serotonin in governing systemic energy metabolism, using animal models with specific serotonin‐related gene knockouts. By inhibiting serotonin's action in the liver and adipose tissues, researchers observed improvements in hepatic steatosis and a reduction in lipid accumulation, respectively (Moon et al., 2022).

The availability of tryptophan (Tph), the synthesis of kynurenine, and the rate‐limiting enzyme for Tph are all directly tied to the synthesis of serotonin. Tph is responsible for the production of the precursor 5‐HTP, which is then rapidly converted into serotonin by the enzyme known as decarboxylase that catalyzes the removal of a carboxyl group (COOH) from an amino acid, leading to the formation of an amine group (NH2) and carbon dioxide (CO2) (Roth et al., 2021). The central and peripheral pools of serotonin are functionally distinct from one another, as they control serotonin‐dependent behaviors in the brain and peripheral areas, respectively. However, serotonin can be absorbed and metabolized in various ways within the body, and its absorption can vary depending on the route of administration. Generally, serotonin is not easily absorbed when taken orally because it has difficulty crossing the blood–brain barrier in its active form (Vicenzi, 2021). Tph predominantly produces circulatory serotonin in the enterochromaffin (EC) cells of the GIT, which are located in the periphery, however, the response of neighboring cells and nutrients control the expression and activity of Tph in EC cells (Haq et al., 2021).

The bulk of ingested tryptophan is used to make kynurenine rather than serotonin. The rate‐limiting enzymes in the conversion of tryptophan to kynurenine are indoleamine 2,3‐dioxygenase (IDO) and tryptophan 2,3‐dioxygenase (TDO) (Yabut et al., 2019). A decrease in serotonin and a rise in kynurenine, associated with depression, have been linked to IDO activity. As a result, the kynurenine pathway, in addition to Tph, is crucial for regulating serotonin synthesis and availability (Songtachalert et al., 2018).

EC cell activation releases serotonin into nearby cells' interstitial space (enterochromaffin cells). EC cells sense postprandial changes in the GI lumen, including pH, nutrition, and toxins. EC cells produce large concentration of serotonin and a tightly regulated regulatory system is needed to remove high serotonin levels from the gut interstitial space, this regulatory system inhibits serotonergic transmission and prevent serotonin poisoning. Serotonin is either circulated or sequestered in enterocytes to remove interstitial serotonin. Enterocytes of the intestinal mucosa take up serotonin via SERT, and monoamine oxidase breaks it down (MAO), then, submucosa capillaries transport serotonin into the bloodstream (Dascalescu & Apetrei, 2021; Prah et al., 2020; Wang et al., 2022).

Classical pathogenesis involves the dysfunction of the serotonergic systems and is mainly reflected by insufficient synaptic 5‐HT levels in the brain. 5‐HT is biosynthesized from dietary L‐tryptophan (Liu, Tian, et al., 2021). However, tryptophan is an essential amino acid critical for the synthesis of serotonin (Gershon, 2013). The synthetic cascade is the same regardless of where in the gut‐brain axis the reaction takes place. Tryptophan is initially converted to 5‐hydroxytryptophan (5‐HTP) by the rate‐limiting enzyme known as tryptophan hydroxylase (TPH). At normal tryptophan concentrations, TPH is not saturated. Consequently, higher tryptophan concentrations can enhance metabolic output. Through aromatic amino acid decarboxylase, the short‐lived 5‐HTP intermediate product is converted into 5‐HT in the next step of the metabolic process (AAAD). On the other hand, the kynurenine pathway is the most important one for tryptophan in terms of its physiological function. After synthesis, in the central nervous system, serotonin is stored in vesicles within serotonergic neurons, ready for release when needed. In the gut, serotonin is stored in enterochromaffin cells (O'Mahony et al., 2015).

After release, serotonin can bind to specific receptors on target cells to transmit its signals. It can also be taken back up into the presynaptic neuron or the enterochromaffin cell by SERTs, a process known as reuptake. Serotonin then undergoes enzymatic breakdown primarily in the liver. The enzyme monoamine oxidase (MAO) converts serotonin into 5‐hydroxyindoleacetic acid (5‐HIAA), which is then excreted in the urine (Von Volkmann et al., 2019).

Serotonin is mostly metabolized through MAO. Both MAO‐A and MAO‐B are isoforms of MAO, with MAO‐A having a far stronger affinity for serotonin. Aldehyde dehydrogenase converts the result of serotonin's MAO‐dependent catabolism, 5‐hydroxyindole aldehyde, into 5‐HIAA (De La Fuente Barrigon, 2018). Thus, serotonin levels are influenced by the availability of tryptophan, TPH expression, and activity, as well as the activity of enzymes involved in serotonin metabolism like MAO, IDO, and TDO.

## 3 ROLE OF SEROTONIN IN CNS AND GI FUNCTIONS

Serotonin plays a pivotal role in both the CNS and GI functions. In the CNS, serotonin functions as a neurotransmitter and is involved in regulating mood, sleep, and appetite. It contributes to feelings of well‐being and helps modulate responses to stress and anxiety, additionally, 5‐HT regulates a wide variety of physiological activities, conventional roles include central regulation of sleep, mood, stress, and anxiety, as well as the peripheral control of GI motility, are among the most well‐known applications of 5‐HT (Jones et al., 2020). 5‐HT is a crucial neurotransmitter in the CNS, which has long been understood as Central 5‐HT. Significant amounts of central 5‐HT are involved in crucial brain processes including mood, sleep, and food regulation (Shajib & Khan, 2015). Since 5‐HT is produced from the mucosa concurrently with peristaltic contractions, serotonin was once assumed to be the primary regulator of peristalsis (Sia et al., 2013).

While there is some evidence pointing to the potential role of the serotonergic system in the pathophysiology of cluster headaches (CH), there has been a notable lack of comprehensive research into serotonin (5HT) metabolism in this context. To address this gap, a study was undertaken to examine the levels of 5HT and 5‐hydroxyindoleacetic acid (5HIAA) in the plasma and platelets of CH patients during active disease episodes. The data from the study indicate that CH is marked by an elevation in plasma serotonergic metabolism, which suggests a potential involvement of the central serotonergic system in the development of CH (D'andrea et al., 1998).

In the GI system, serotonin is mainly produced in enterochromaffin cells (ECs) of the gut lining. It influences various aspects of gut function, including peristalsis (intestinal muscle contractions), secretion of digestive enzymes, and sensory perception related to the digestive process. Additionally, serotonin in the gut plays a role in regulating appetite and satiety (Habets et al., 2013). Serotonin regulates gut motility by influencing the contractions of intestinal muscles. It helps maintain the rhythmic movements necessary for the proper digestion and absorption of nutrients (Borgdorff & Tangelder, 2012). Serotonin receptors in the gut are involved in sensory perception related to the digestive process. They contribute to the perception of fullness and discomfort, influencing eating behaviors, also they can stimulate the secretion of digestive enzymes, aiding in the breakdown of food in the stomach and small intestine (Brommage, 2015).

Moreover, serotonin has been involved in a wide variety of processes that take place inside the human body. Since its connection between the brain and the gut (the brain‐gut axis), as well as the growing clinical evidence indicating the efficacy of SSRIs (Selective serotonin reuptake inhibitors) in treating these neurological disorders/conditions, it has been demonstrated that serotonin and its receptors, specifically the SERT and its polymorphisms, play a potential role in the pathophysiology of functional digestive disorders such as irritable bowel syndrome (IBS) (Margolis, 2017; Wang et al., 2004; Yaghoubfar et al., 2020). Serotonin plays a key role in secretion, vasodilation, peristalsis, perception of pain, and nausea through the 5‐HT receptors in the GIT. Tryptophan from the GIT can enter circulation, cross the blood–brain barrier (BBB), and initiate serotonin synthesis in the brain (Westfall et al., 2017).

There is mounting evidence that points to 5‐HT in the periphery playing a significant role as a hormone able to modulate metabolic processes in the periphery (Martin et al., 2017). 5‐HT also upregulates hepatic gluconeogenesis and adipocyte lipolysis during fasting. However, fasting increases intestinal Tph1 expression and circulating 5‐HT, like glucagon (Fu et al., 2016). Contrary to common belief, obesogenic circumstances also result in higher circulating levels of 5‐HT. Obese people have higher quantities of the hormone 5‐HT in their blood and intestines (Young et al., 2018). Moreover, peripheral 5‐HT regulates glucose‐stimulated insulin release in islets, and excessive levels of peripheral 5‐HT produce hepatic steatosis, an increase in liver fat (Zhang et al., 2017).

**FIGURE 1:**
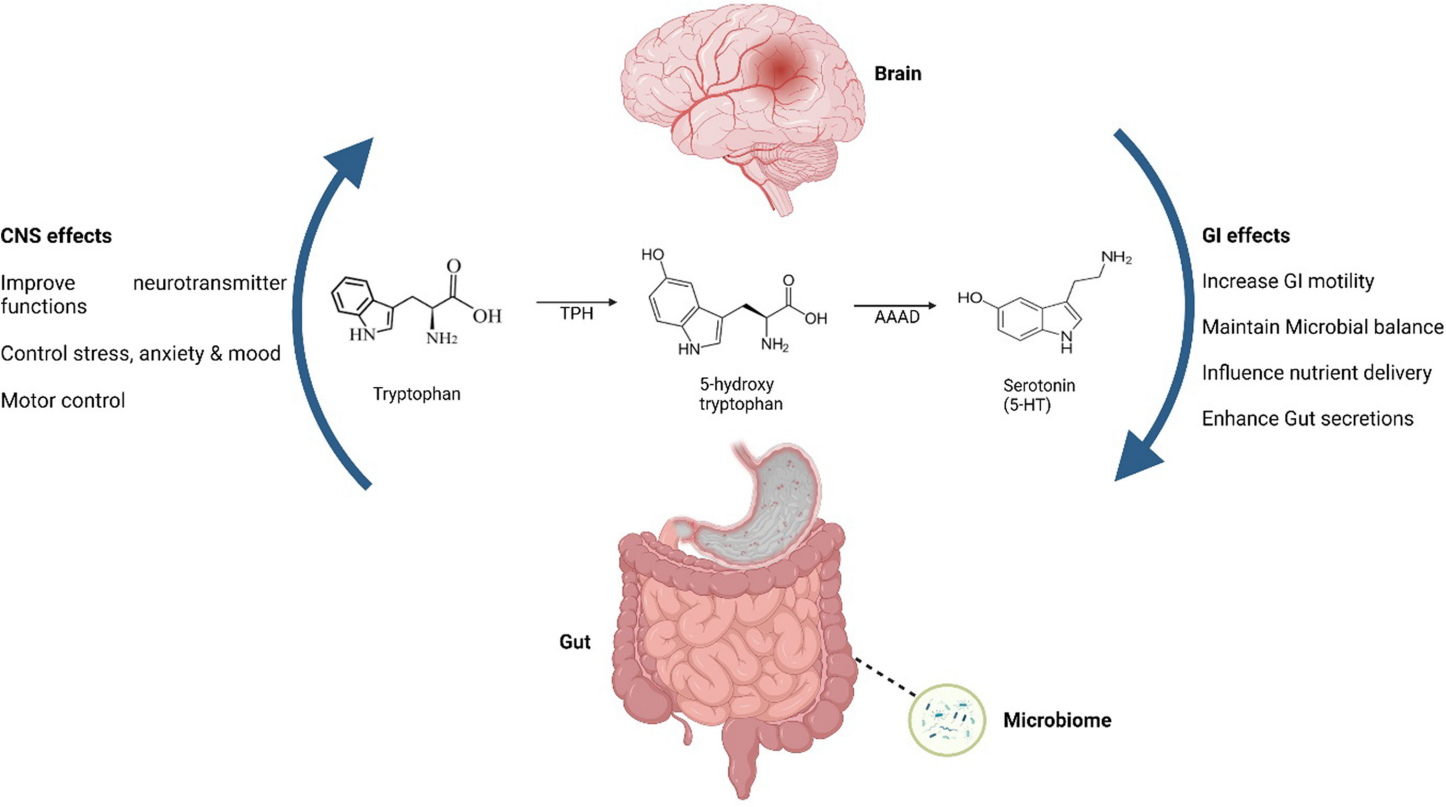
Effects of serotonin‐5HT on central nervous system & GI tract functions.

Because 5‐HT is a vital mediator of many important gut functions and CNS activities, as a consequence, a relevant target in the setting of GI disorders. However, Figure 1 shows the graphical presentation of the interplay between CNS, GIT, and serotonin signaling.

## 4 SEROTONIN AND GUT–BRAIN AXIS

The gastrointestinal tract and the central nervous system are connected by a communication pathway known as the brain‐gut axis. This pathway allows for information to flow in both directions. At both the beginning and the end of this network, serotonin plays a critical role as a neurotransmitter. There is an increasing body of research that points to the gut microbiome playing an important role in the regulation of the typical functioning of this axis. The enterochromaffin cells (ECCs) in the gastrointestinal system are responsible for the production of around 90% of the body's total serotonin. The brain only contains approximately 5% of the remaining serotonin (O'Mahony et al., 2015; Yano et al., 2015).

The hypothalamic–pituitary–adrenal (HPA) axis is the major connection that exists between the gastrointestinal tract and the brain. The bacterium in the gut can control the levels of stress hormones such as cortisol through this axis. The gastrointestinal tract's ability to activate stress circuits is accomplished through vagal pathways, which are also involved in the activation and regulation of the HPA axis (Foster & Neufeld, 2013).

This microbiota–gut–brain axis, also known as the MGBA, is essential for normal development and function, and abnormalities in its control have been linked to a variety of neurological and gastrointestinal conditions. However, serotonin has been discovered as a chemical of particular importance as a result of research into the molecular underpinnings of the MGBA (Everett et al., 2022).

A study evaluated the effects of *Akkermansia muciniphila* and its extracellular vehicles (EVs) on genes pertaining to the serotonergic system in the colon and hippocampus of mice, *Akkermansia muciniphila* was intervened for 4 weeks, and the serotonin levels in the colon, hippocampus, and serum of mice, as well as the human colon carcinoma cells (Caco‐2), were measured by ELISAs. Results revealed that serotonin signaling/metabolism through the gut–brain axis might be considered in new therapeutic strategies to ameliorate serotonin‐related disorders (Yaghoubfar et al., 2020).

In a recent study, derived *Bifidobacterium dentium* has been utilized to modulate the mammalian serotonergic levels and gut–brain axis, results revealed increased fecal acetate in the mice associated with *Bifidobacterium dentium*, findings have suggested that *B dentium*, and the bacterial metabolite acetate, are capable of regulating key components of the serotonergic system in multiple host tissues, and are associated with a functional change in adult behavior (Engevik, Luck, et al., 2021).

However, the response of stress hormones that are produced down the HPA axis can be reversed using probiotics, which are live microorganisms that are taken as dietary supplements or food products to improve health (Liu et al., 2019). In addition to other neuroanatomical abnormalities in levels of neurotransmitters, it has been discovered that abnormalities of the HPA axis, as well as hyperactivity of the HPA axis, may be potential biological contributors to the development of anxiety and depression (i.e., chemical substances that deliver hormonal responses in the brain). There is a connection between depression and the HPA axis because bouts of depression are associated with a dysregulation of this axis, and the resolution of depressive episodes is connected with normalization of this axis. Both in the early stages of development, when the programming of the HPA axis is being done, and throughout life, when a person's response to stress is being determined, the microbiota in the gut plays a significant role (Wallace & Milev, 2017). Table 2 shows the systematic review of probiotic supplementation improves serotonin signaling via the gut–brain axis.

## 5 GUT MICROBIOTA REGULATE SEROTONIN TRANSMISSION

The gut microbiota can influence serotonin levels in the brain and neurotransmission through various mechanisms, including serotonin production in the gut, modulation of the SERT, immune system interactions, vagus nerve communication, and the production of metabolites like SCFAs, however, gut bacteria ferment dietary fibers to produce short‐chain fatty acids, such as butyrate, propionate, and acetate. These SCFAs can have far‐reaching effects on the brain and neurotransmission (Borgdorff & Tangelder, 2012; Margolis, 2017).

Numerous bacteria that are present in the gut have an impact on its motility and functionality. The complex of microbes that live in the gut and have a symbiotic interaction with the host is known as the intestinal microbiota (Malard et al., 2021). The term “microbiome” refers to the aggregate gene pool of the bacteria that share this particular ecological niche. The number of species in this microbiota grows from the esophagus to the rectum, where it is not spread equally along the gut (Mailhe et al., 2018). From infancy through adulthood, the gut microbiota's makeup is extremely dynamic and varies. Bacteria are categorized into phylum, order, families, genera, and species (Ugwu et al., 2020).

The composition of the intestinal microbiota can change over the course of adulthood due to a variety of factors, which can then have an impact on one's health or propensity for disease (Segal et al., 2020). It has been demonstrated that “normal” intestinal microbiota contributes to a variety of physiological advantages for the host, including control of intestinal homeostasis, defense against pathogens, immune system development, and metabolic and neuroendocrine processes (Ma & Ma, 2019). However, dysbiosis, a change in the intestinal microbiota, is acknowledged as a risk factor for the beginning and development of several chronic illnesses, particularly those connected to metabolic alterations, including non‐alcoholic fatty liver disease (NAFLD) (Vijay & Valdes, 2022). However, enteroendocrine cells can be triggered to respond in a sensory manner by ingested nutrients, microbial metabolites, immunological activation, and some medicines. It is common knowledge that enteroendocrine cells perform the function of sensory cells and are capable of reacting to their immediate surroundings (Findeisen et al., 2019; Lund et al., 2018; Sun et al., 2019).

It is widely established that 5‐HT plays an important part in the control of gastrointestinal function. Nevertheless, the involvement of gut microorganisms in altering host gut‐derived 5‐HT signaling is a developing subject that has the potential to yield crucial insights into the relationship between the microbiota and GI function (Spear & Mawe, 2019).

Numerous studies looking at the function of the microbiota in gut‐derived 5‐HT regulation found that GF mice have significantly lower serum 5‐HT levels, decreased colonic Tph1 mRNA expression, and increased colonic SERT mRNA expression as compared to control mice, even though it has long been known that the enteric community of bacteria that inhabits the human distal intestinal track has a significant impact on health (Liu et al., 2016; Tada et al., 2016).

A recent study explored the relationship between the neurotransmitter serotonin (5‐HT), its transporter (5‐HTT), early life stress, and gut microbiota composition in rats. The study investigates whether alterations in 5‐HTT expression, particularly in rats with the 5‐HTT‐linked polymorphic region (5‐HTTLPR) short allele, are associated with changes in gut microbiota and how these changes may be influenced by early life stress. The findings of the study suggested that the 5‐HTT genotype had a more pronounced impact on microbial dysbiosis. Rats with the 5‐HTT knockout genotype exhibited a significant difference in microbiota composition compared to the other genotypes. However, the findings may have implications for understanding similar relationships in humans and the potential role of gut microbiota in intestinal and psychiatric disorders associated with serotonin dysfunction (El Aidy et al., 2017).

The importance of the gut microbiome in regulating GI function is becoming more widely recognized. Diseases with altered GI motility have been linked to altered gut microbiota composition. A recent study examined germ‐free (GF) or humanized mice to establish the link between gut bacteria, colonic contractility, and host serotonergic gene expression (with human gut microbiota). Both GF and HM colons had shorter contractile times after 5‐HT treatment. Short‐chain fatty acids (SCFAs) enhanced TPH1 transcription in a human EC cell culture, according to the study. Consequently, it has been established that enteric 5‐HT synthesis and homeostasis are significantly influenced by the gut microbiota acting through SCFAs (Reigstad et al., 2015). A study showed that the microbiota is essential for controlling host 5‐HT, the study investigates that Indigenous spore‐forming bacteria (Sp) from the mouse and human microbiota promote 5‐HT biosynthesis from colonic enterochromaffin cells (ECs), moreover, gut bacteria metabolize tryptophan into various compounds, some of which may ultimately contribute to serotonin production which provides 5‐HT to the mucosa, lumen, and circulating platelets, could encourage to produce 5‐HT humans (Yano et al., 2015).

An investigation was conducted to investigate the host‐microbe interrelationships that govern peripheral 5‐HT by analyzing the influences that microbes have on the fecal metabolome. In comparison to particular pathogen‐free controls, the researchers discovered that GF mice had significantly lower amounts of 5‐HT in both their colonic and feces contents. This lack of 5‐HT was seen in the distal, middle, and proximal colon, but not the small intestine, suggesting that bacteria regulate the quantity of 5‐HT in the colon. In this work, there was no discernible difference between the quantity of chromogranin A (CgA+) ECs in the colons of adult GF mice and SPF mice. This suggests that a decrease in colon 5‐HT may be due to an aberrant 5‐HT metabolism rather than an impaired production of ECs (Liu et al., 2021; Yano et al., 2015).

Recent data is consistent with the gut microbiota playing a causal role in regulating gut 5‐HT levels and host metabolism. As a result, gut‐derived 5‐HT plays a crucial role in encouraging long‐term energy conservation and enhancing numerous physiological responses to nutrient deprivation.

## 6 SEROTONIN AND GASTROINTESTINAL DISORDERS

As the GI tract is the body's primary source of serotonin (5‐hydroxytryptamine (5‐HT)), its function and physiology are inextricably linked to that of the GIT. Inflammatory bowel illness and functional disorders like irritable bowel syndrome have been linked to 5‐HT, which is produced by enterochromaffin (EC) cells and is an important enteric mucosal signaling molecule. 5‐HT has also been implicated in several other GI diseases. 5‐HT can play a variety of functions in the pathophysiology of gastrointestinal illnesses; however, two of the most significant roles for 5‐HT are its ability to control the immune system and its influence on the motility of the gastrointestinal tract (Guzel & Mirowska‐Guzel, 2022). Moreover, various serotonin receptors have been presented in Table 1 with respect to their role in the GIT.

**TABLE 1:** Function of serotonin receptors in GI tract.

Receptor	Function in GI	Reference
5‐HT1	Gastric fundus relaxation	Guzel and Mirowska‐Guzel (2022)
	Contraction of intestinal circular muscle	Fujii et al. (2020)
	Contraction of intestinal longitudinal muscle	Gwynne and Bornstein (2019)
	Prokinetic intestinal stimulation	Legan et al. (2022)
	Peristaltic and secretory reflexes	Brown and Liu (2021)
5‐HT2	Contraction of smooth muscles	Guevara and Trejo (2021)
	Contraction of smooth muscles in stomach fundus	Li, Wang, et al. (2019)
	Relaxation of longitudinal muscle in the intestine	Tonini and De Ponti (2020)
5‐HT3	Chloride secretion and serotonin release from EC cells	Spencer and Keating (2022)
5‐HT4	Increase intestinal motility	Shokrollahi et al. (2019)
	Relaxation of colon	Li, Wang, et al. (2019)

### 6.1 Inflammatory bowel syndrome

Irritable bowel syndrome (IBS) is a chronic disorder that is widespread, incompletely known, and characterized by stomach discomfort linked with abnormal bowel movements. This condition occurs even in the absence of anatomical or biochemical abnormalities in the intestines. It is a complicated condition that is related to alterations in the movement, excretion, and sensation of the gastrointestinal tract. In the gastrointestinal system, serotonin (5‐HT) functions both as a neurotransmitter and as a molecule that is involved in paracrine signaling. Initiation of peristaltic, secretory, vasodilatory, vagal, and nociceptive reflexes is caused by the release of 5‐HT from enterochromaffin (EC) cells. Irritable bowel syndrome is characterized by changes in 5‐HT signaling, which may be the cause of both intestinal and extraintestinal symptoms (Pretorius & Smith, 2020).

Both the enterochromaffin (EC) subtype of enteroendocrine cells and the serotonergic neurons in the myenteric plexus are responsible for the synthesis of serotonin within the colon itself. In response to luminal stimuli, such as mechanical stresses, EC cells will release 5‐HT into the surrounding environment (Kuramoto et al., 2021). 5‐HT is a neurotransmitter that is produced and then acts on receptors in the processes of sensory neurons that enter the lamina propria. These include primary sensory neurons with cell bodies in the spinal (dorsal root) and vagal (nodose) ganglia, as well as branches of intrinsic sensory neurons with cell bodies in the submucosal and myenteric ganglia. Thus, the activation of motor, secretory, and vasodilatory reflexes as well as the stimulation of afferent signals to the brain and spinal cord are brought on by the release of 5‐HT from EC cells (Bosi et al., 2020).

Constipation‐predominant IBS (also known as C‐IBS), diarrhea‐predominant IBS (also known as D‐IBS), and alternating forms are the three most common classifications of irritable bowel syndrome (IBS). Recent research suggests that variations in serotonin (5‐HT) signaling may play a role in functional gastrointestinal diseases such as irritable bowel syndrome (IBS), chronic constipation, diarrhea, and functional dyspepsia. There have been reports of changes in enteroendocrine cells in patients with ‘post dysenteric’ IBS, which is characterized by recurring stomach pain and diarrhea (Ng et al., 2018).

Researchers found that a decrease in intestinal serotonin production causes the intestinal lining to weaken, which inevitably causes obstruction or constipation and an increase in serotonin levels in the gut (Bruta & Bhasin, 2021). It has been found that people with IBS have enterocytes that lack the SERT. However, many studies focus on the lower density of enterochromaffin cells in the GIT of IBS sufferers. It has been demonstrated that indoleamine pyrrole 2,3‐dioxygenase overexpression activates a metabolic pathway that is probably related to the development of IBS. After that, research has revealed that IBS patients—both men and women—have higher kynurenine concentrations than normal. Consequently, a favorable correlation between IBS and the degree of kynurenine/tryptophan was discovered (Yeung et al., 2022). However, tryptophan is longer shunted via the kynurenine pathway in people with extreme IBS side effects, which helps explain the atypical serotonergic action. Reduced stool production and delayed upper GIT motility have been observed in mice given paroxetine, an SSRI, over an extended period (Bruta & Bhasin, 2021). These discoveries propose that changed mucosal 5‐HT signaling seems to contribute to the indications of IBS.

### 6.2 Functional dyspepsia

Functional dyspepsia, often known as FD, is a prevalent digestive illness that impacts between 20 and 30 percent of the adult population all over the world. Chronic recurrent epigastric symptoms, such as discomfort, burning, postprandial fullness, and early satiation, are a hallmark of this condition (Wauters et al., 2021).

The meta‐analysis and the systematic review have shown that there was a large overlap between FD and IBS, with 37% of patients diagnosed with dyspepsia also suffering from IBS at the same time (De Bortoli et al., 2018). Additionally, abnormalities of 5‐HT were detected in patients who had both postinfectious IBS and FD at the same time. On the other hand, there is a paucity of data regarding the anomalies of 5‐HT in people who have FD. It has been suggested that agonists of the 5‐HT4 receptor may alleviate dyspeptic symptoms, particularly in patients whose stomach emptying is delayed (Sato & Grover, 2022).

### 6.3 Celiac disease

Celiac disease, also known simply as celiac, is an immunological reaction to gluten that causes patients to suffer from persistent diarrhea and extreme exhaustion. Celiac disease has been linked to an increase in both the number of enterochromaffin cells (EC) and the quantity of 5‐hydroxytryptamine (5‐HT) in the duodenal mucosa, as well as crypt hyperplasia in the small intestine. The presence of an increased number of neuroendocrine cells that produce 5‐hydroxytryptamine (5‐HT) in the mucosa of patients with refractory celiac disease is suggestive of a role for 5‐HT in maintaining an inflammatory response in these patients. Although additional research is required to determine the exact role that 5‐HT plays in the pathogenesis of celiac disease, it is now abundantly clear that 5‐HT has a strong correlation with the onset of celiac disease (Banskota et al., 2019).

## 7 SEROTONIN AND BRAIN DISORDERS

Serotonin, often referred to as the “feel‐good” neurotransmitter, plays a crucial role in the intricate workings of the human brain and its impact on our emotional well‐being, the neurotransmitter is involved in a wide range of physiological and psychological processes, making it a subject of intense scientific inquiry and a key player in understanding various brain disorders (Awasthi & Chandra, 2021). The intricate relationship between serotonin and brain disorders is a fascinating and complex one, offering insights into both the causes and potential prevention of neurological and mental health conditions.

Serotonin, primarily synthesized in the neurons of the brainstem and found throughout the central nervous system, serves as a vital communicator between nerve cells. Its influence extends far beyond regulating mood, serotonin is involved in controlling sleep, appetite, digestion, and even cardiovascular functions (O'Mahony et al., 2015). This widespread presence underscores its importance in maintaining overall brain and body health.

Serotonin is often associated with mood regulation, and imbalances in serotonin levels are linked to depression, while serotonin itself may not prevent depression, however, maintaining adequate levels of this neurotransmitter can help stabilize mood and reduce the risk of developing depressive disorders, many antidepressant medications work by increasing serotonin levels in the brain (Martins & Brijesh, 2018; Naoi et al., 2018).

Additionally, anxiety disorders, such as generalized anxiety disorder and panic disorder, are also influenced by serotonin levels, adequate serotonin signaling can help regulate anxiety, reducing the likelihood and severity of anxiety‐related disorders (Guilmette, 2022).

Serotonin plays a role in regulating blood vessel flow, however, imbalances in serotonin levels can lead to blood vessel constriction that may potentially trigger migraines, although, proper serotonin signaling can help prevent migraine attacks or reduce their frequency and intensity (Körtési et al., 2022).

Serotonin dysregulation can contribute to the development of Obsessive‐compulsive disorder (OCD), moreover, medications that increase serotonin levels, such as selective serotonin reuptake inhibitors (SSRIs), are often used in the treatment of OCD and can help prevent its progression (Hadi et al., 2021). Serotonin has been shown to be involved in regulating appetite and satiety. Imbalances in serotonin signaling can contribute to eating disorders such as anorexia nervosa and bulimia nervosa. Moreover, maintaining a healthy serotonin balance can play a role in preventing these conditions (Prochazkova et al., 2023).

While not directly preventing neurodegenerative diseases like Alzheimer's and Parkinson's, serotonin signaling has been linked to cognitive function and neuroprotection. Adequate serotonin levels may support brain health and potentially delay the onset or progression of these disorders (Gupta et al., 2023). A study investigated that *Saccharomyces boulardii* CNCM I‐1079 may be distinct from bacterial probiotics in its salivary serotonergic effect, which appears positively linked to sympathoadrenal markers (Karbownik et al., 2022). Understanding these complexities is essential for the development of targeted therapies and interventions that may help prevent or alleviate the burden of brain disorders on individuals and society as a whole.

## 8 PROBIOTICS PROMOTE SEROTONIN SIGNALING

Oral administration of isolated bacterial strains, such as those present in non‐colonizing “probiotic” formulations, is a short‐term method of modifying the gut microbiota. Orally administered microorganisms have been shown in numerous studies to have positive impacts on host physiological processes in both animal models and humans (Kim et al., 2017).

Serotonin (5‐hydroxytryptamine [5‐HT]) generation by enterochromaffin cells can be controlled by the microbiota in the human gut. So far, the mechanism underpinning serotonin signaling caused by microbes is not fully known. A recent study evaluated the impact of B dentium metabolites on enterochromaffin cells' production of 5‐HT. In this investigation, steroids from mice and humans were employed. Mature germ‐free mice were treated with sterile media, live Bifidobacterium dentium, heat‐killed B dentium, or live Bacteroides ovatus. These findings imply that both the bacterial metabolite acetate and the bacterial strain are capable of altering the function of adult behavior and controlling important serotonergic system components in a variety of host tissues (Engevik, Luck, et al., 2021).

According to numerous research, probiotic strains including *Lacticaseibacillus rhamnosus* and *Limosilactobacillus reuteri* are linked to higher intestine SERT concentrations (Engevik, Ruan, et al., 2021; Wang et al., 2015). Irritable bowel syndrome (IBS) can be relieved in part by probiotics; however, the exact mechanism is unknown. Researchers have looked into how the supernatants of the bacteria *Lactobacillus acidophilus* and *Bifidobacterium longum* affect the expression of the protein and messenger ribonucleic acid (mRNA) for the SERT (Cao et al., 2018). A research carried out by Li, Liu, et al. (2019), investigated to examine the potential antidepressant properties of prebiotics and probiotics and to investigate their impact on the modulation of serotonin (5‐HT) metabolism. The results suggested that both prebiotics and probiotics exhibit antidepressant effects and exert a notable influence on the regulation of 5‐HT metabolism, with particular emphasis on the role of *L. rhamnosus*.

The bacteria in the intestines have the potential to influence the amount of serotonin found in the gastrointestinal system. Through neurological processes that take place between the ENS and the central nervous system, it is also able to alter the serotonergic neurotransmission of the host in the gut‐brain axis (CNS) (Yaghoubfar et al., 2020). Inflammatory bowel disease (IBD) and mental illnesses, such as mood disorders, anxiety disorders, depression, and other systemic disorders, can be caused by a dysbiosis of the gut microbiota and dysfunction of the serotonergic system (Yong et al., 2020). There is a connection between the microbiota in the stomach and the mucosal, neuronal, and systemic homeostasis of the neurotransmitter serotonin. Therefore, altering the gut microbiota with probiotics can help maintain the homeostasis of the serotonin system and avoid several pathophysiological diseases in the host (Yaghoubfar et al., 2020). Altogether, the mounting evidence suggests that probiotics can alter host gut‐derived 5‐HT and influence 5‐HT‐mediated gut functions, leading to potential therapeutic applications. Table 2, shows the systematic review of probiotic supplementation improves serotonin signaling via gut‐brain axis.

**TABLE 2:** Probiotic supplementation improves serotonin signaling.

Probiotic	Study design	Duration	Targeted organ	Objective	Key findings	Reference
*Lactobacilli*	Mice study	8 weeks	Gut	Probiotics reduce inflammation in Multiple Sclerosis (MS)	The serotonin gene expression increased	Sajedi et al. (2021)
*L. plantarum*	Mice‐model	14 days	Brain	Probiotics improve mental health via gut‐brain axis	Promotes serotonin signaling, intestinal motility & mucin production	Chen et al. (2022)
*Saccharomyces boulardii*	Mice study	–	Gut	Probiotics regulate Intestinal serotonin transporter	Prevent IBS and diarrhea, upregulate SERT & inhibits gut motility	Gu et al. (2022)
*Bifidobacterium animalis*	In vitro	–	Gut	Probiotics enhance GI motility in Zebrafish	Increased intestinal peristalsis and modulation of serotonin	Lu et al. (2019)
*Streptococcus* & *Lactobacillus strains*	Rat study	25 days	Brain and Gut	Probiotics enhance cross talk among serotonin receptors and gut	Control memory deficit & increase serotonin receptors	Beilharz et al. (2018)
*Probiotic strains*	In vivo model	–	Brain	Probiotics modulate serotonin signaling	Improves pathophysiological disorder	Mahesh et al. (2021)
*Limosilactobacillus reuteri*	Mice model	–	Gut	Probiotics upregulate serotonin signaling	Upregulation of SERT to maintain intestinal homeostasis	Engevik, Ruan, et al. (2021)

## 9 CONCLUSION

In recent years, there has been growing interest in the role of the gut microbiota and the impact it has on our overall health. One area of particular interest is the relationship between the gut microbiota and the brain, known as the gut‐brain axis. This connection is thought to be mediated by the production and signaling of various neurotransmitters, including serotonin.

Research has shown that probiotics, which are live microorganisms that confer health benefits when consumed, can have a positive impact on gut health. Studies have found that probiotics can increase serotonin levels in the gut, which can lead to improvements in gut motility, immune function, and even mood.

However, it is important to note that the relationship between probiotics, serotonin, and gut health is complex and strongly interlinked. Probiotics in gut microbiota may play a significant role in promoting serotonin signaling and improving gut health. Additionally, more research is needed to determine the long‐term effects of probiotic use and its impact on overall health.

## AUTHOR CONTRIBUTIONS

**Noor Akram:** Conceptualization (equal); writing – original draft (equal); writing – review and editing (equal). **Zargham Faisal:** Validation (equal); writing – original draft (equal); writing – review and editing (equal). **Rushba Irfan:** Software (equal); validation (equal); writing – review and editing (equal). **Yasir Abbas Shah:** Writing – original draft (equal); writing – review and editing (equal). **Syeda Ayesha Batool:** Visualization (equal); writing – review and editing (equal). **Toobaa Zahid:** Formal analysis (equal); validation (equal); writing – review and editing (equal). **Aqsa Zulfiqar:** Formal analysis (equal); validation (equal). **Areeja Fatima:** Data curation (equal); formal analysis (equal). **Qudsia Jahan:** Data curation (equal); validation (equal); visualization (equal). **Hira Tariq:** Formal analysis (equal); validation (equal). **Farhan Saeed:** Conceptualization (equal); writing – review and editing (equal). **Aftab Ahmed:** Data curation (equal); validation (equal); writing – review and editing (equal). **Aasma Asghar:** Formal analysis (equal); writing – review and editing (equal). **Huda Ateeq:** Formal analysis (equal); validation (equal). **Muhammad Afzaal:** Supervision (equal); writing – original draft (equal); writing – review and editing (equal). **Mahbubur Rahman Khan:** Validation (equal); writing – review and editing (equal).

## FUNDING INFORMATION

The authors declare that no funds, grants, or other support were received during the preparation of this manuscript.

## CONFLICT OF INTEREST STATEMENT

The authors declare that they have no known competing financial interests or personal relationships that could have appeared to influence the work reported in this paper.

## Data Availability

Even though adequate data has been given in the form of tables and figures, however, all authors declare that if more data is required then the data will be provided on a request basis.
